# Patterns of Communication About Serious Illness in the Years, Months, and Days before Death

**DOI:** 10.1089/pmr.2022.0024

**Published:** 2022-08-04

**Authors:** Mattias Tranberg, Juliet Jacobsen, Carl Johan Fürst, Jacob Engellau, Maria E.C. Schelin

**Affiliations:** ^1^ Division of Palliative Care, Department of Clinical Sciences Lund, Lund University, Lund, Sweden.; ^2^ The Institute for Palliative Care at Lund University and Region Skåne, Lund, Sweden.; ^3^ Department of Palliative Care and Geriatric Medicine, Massachusetts General Hospital, Boston, Massachusetts, USA.; ^4^ Harvard Medical School, Boston, Massachusetts, USA.; ^5^ Department of Oncology Hematology and Radiophysics, Skåne University Hospital, Lund, Sweden.

**Keywords:** communication, end of life, patients, physicians, serious illness, transition

## Abstract

**Background:**

Communication with patients and families about serious illness impacts quality of life and helps facilitate decision-making.

**Objective:**

To elucidate the pattern of communication about serious illness for patients who have died in an inpatient setting.

**Design:**

Three hundred patients from the Swedish Registry of Palliative Care 2015–2017 were randomly selected for manual chart review.

**Setting:**

Patients who died in a palliative care, oncology, or internal medicine unit in Sweden were selected.

**Measurements:**

We report on the frequency of conversations at three time points, 6 months or longer before death (“Years”), 15 days–6 months before death (“Months”), and 0–14 days before death (“Days”). We also report the timing of the conversation about dying.

**Results:**

A total of 249 patients were included after exclusions; they had an average of 2.1 conversations (range 1–6). The first conversation took place a median of 53 days before death and the last conversation took place a median of 9 days before death. Separate conversations with the next of kin took place a median of two days before death. We could verify a conversation about dying in only 156/249 (63%) medical records.

**Conclusions:**

Communication about serious illness between clinicians, patients, and families occurs iteratively over a period before death. Measuring the quality of communication about serious illness using a years, months, and days framework may help ensure that patients and families have sufficient information for medical and personal decision making.

## Introduction

Communication about serious illness is important throughout the disease trajectory. In the years before death, many patients want to pursue treatment for the possibility of more time. Communication is less concerned with treatment decision making, as the goals of medical care are often straightforward, and instead focuses on understanding patients' values and goals for living, and is increasingly recognized as an important way to lessen patients' anxiety and depression^1^ and improve quality of life.^2^

In the months before death, as patients become sicker, they encounter more complex decisions related to treatment options and how to balance a decreasing potential for treatment benefit with hope to live longer or with an improved or sustained quality of life. Communication ideally focuses on prognosis, which is described by patients and their next of kin as important for planning and preparation, and on balancing competing goals.^3^ Toward the end of life, patients and families face decisions about dying, and communication that explicitly or implicitly recognizes the dying process is a near universal component of care.^4^

To support and encourage communication toward the very end of life, the Swedish “National Guideline for Palliative Care at the End Of Life” highlights the importance of communicating with patients or their next of kin. The guidelines require Swedish clinicians to have and document a conversation about dying, defined as “informative conversation about the transition to end-of-life care.”^5^ In these conversations, the clinician, patient, and their next of kin together discuss that the goals of care are quality-of-life and symptom management, rather than extension of life. The conversations are encouraged to be reported to the Swedish Register for Palliative Care (SRPC). Reporting can be done by any clinician and does not imply that specialist palliative care has been provided.

In the region of Skåne, where this study was performed, the rate of documentation of conversations in the SRPC was 32% at the start of our study (2015), which is far from the target of 98%.^6^ Little is known about when conversations occur during an illness trajectory, or even if patients have more than one. To better understand the process of communication about serious illness and the experiences of patients and families, we performed a medical record review of all communication about serious illness from diagnosis of the main disease until death.

## Materials and Methods

The SRPC is a unique registry that records quality measures on terminal care irrespective of where care is provided. From this registry, we randomly selected 300 (46%) of 543 individuals who died 2015–2017 in three specific wards: specialized palliative care, oncology, or patients diagnosed with chronic obstructive pulmonary disease (COPD) in internal medicine in the region of Skåne. All those selected had been inpatients in the three wards and were reported to the SRPC as having had a conversation about dying, either directly with the patient or with their next of kin. Patients who had lost the ability to express their will more than a month before the time of death, or who were at the internal medicine ward, but were not diagnosed with COPD were excluded.

All medical records were examined by the first author and a research assistant separately according to the following protocol: first, medical records were electronically searched for the term “conversation about dying.” Then, both assessors reviewed the medical records to identify any documentation related to communication about serious illness, including conversations about prognosis, goals of care, palliative care, and dying. Furthermore, the patients' medical records were also read in their entirety from the time of diagnosis of the main disease (e.g., diagnosis of cancer, diagnosis of COPD) until death, to identify other record entries of conversations about serious illness or similar and to examine conversations that had been documented over time, including those at previous care services and institutions. Continuous discussions between the first author and research assistant regarding content interpretation were held to ensure consistency in the analyses.

**FIG. 1.**
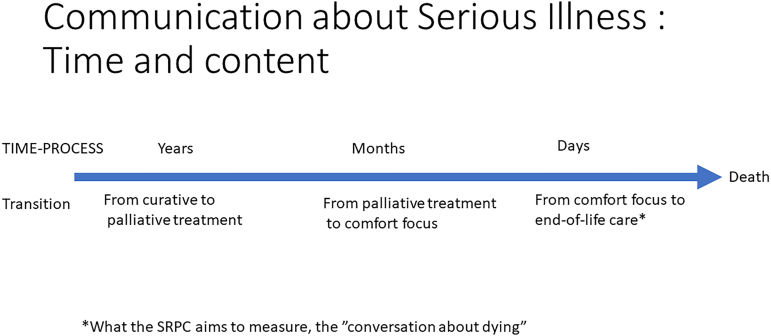
A time-related framework to help identify transitions and conversations in a palliative care and disease trajectory.

Documentation was reviewed to identify three transitions: the transition from curative to palliative treatment intent, the transition from palliative treatment to a focus on comfort, and discussions about dying (Fig. 1). Documented conversations about related topics such as code status were not classified as conversations about dying, that is, we did not infer the conversation content. We recorded the first documented conversation about dying with the patient, and further conversations with the patient reiterating or elaborating on this message were not recorded.

### Analysis

We used descriptive statistics to report the characteristics of the population and compare data from medical records with data from the SRPC. (SAS Enterprise Guide version 8.1; SAS Institute, Cary, NC). We reported the median time from the conversations until death.

Conversations were grouped according to a previously described years, months, and days framework.^7^ The cutoff between years and months was chosen at six months before death, because this time point roughly reflects the time period when clinical care shifts from treatment focused on the disease to treatment focused on comfort, and aligns with the Medicare hospice eligibility requirements in the United States (based on the clinicians best estimate of the prognosis). The cutoff between months and days was chosen at 14 days before death because this time point roughly reflects the start of the dying process.^8^

### Ethics

This was a retrospective review of medical records and registered data of deceased subjects; thus, no informed consent was obtained, in accordance with Swedish legislation and ethical approval for this study. This study was approved by the Regional Ethical Review Authority of Lund University (ref. 2018/608).

## Results

### Patients

Of the 300 patients, 42 (14%) were excluded because they had lost the ability to express their will more than one month before death or had mistakenly been identified as having COPD as the cause of death. Nine patients had no entry regarding communication about serious illness in the medical records, despite SRPC reporting them as having had at least one; thus, 249 patients were included in the analysis. The average age of the patients was 72 years (range, 32–94 years), and 61% were women (Table 1). Most patients (160/249) died in the specialized palliative care unit.

**Table 1.** Basic Demographics

	*N* (%)
Age	
32–49	12/249 (5%)
50–59	25/249 (10%)
60–69	56/249 (22%)
70–79	92/249 (37%)
80–89	47/249 (19%)
90–94	17/249 (7%)
Sex	
Female	149/249 (60%)
Male	100/249 (40%)
Place of death	
Specialized palliative care	160/249 (64%)
Oncology ward	49/249 (20%)
COPD at Internal Medicine ward	40/249 (16%)
No. of conversations	513
In the years window	33/513 (6%)
In the months window	201/513 (39%)
In the days window	279/513 (54%)

COPD, chronic obstructive pulmonary disease.

^*^ Correction added on September 12, 2022 after first online publication of August 1, 2022: In Table 1, N (%) for age 90-94 was mistakenly noted as 1/2497 (7%). The table has been corrected to reflect 17/249 (7%).

### Timing of conversations

From the time of diagnosis of serious illness until death, patients had an average of 2.1 (range 1–6) conversations regarding prognosis, goals of care, palliative care, and dying. The median time for the first conversation was 53 days before death and the median time for the last conversation was 9 days before death. Eighty-eight patients had only one conversation documented in their medical records at a median of 16 days before death. Conversations were most frequently held during the last two weeks of life, the “days window” (Table 1). Separate conversations with their next of kin (*n* = 137) were reported at a median of two days before death.

**FIG. 2.**
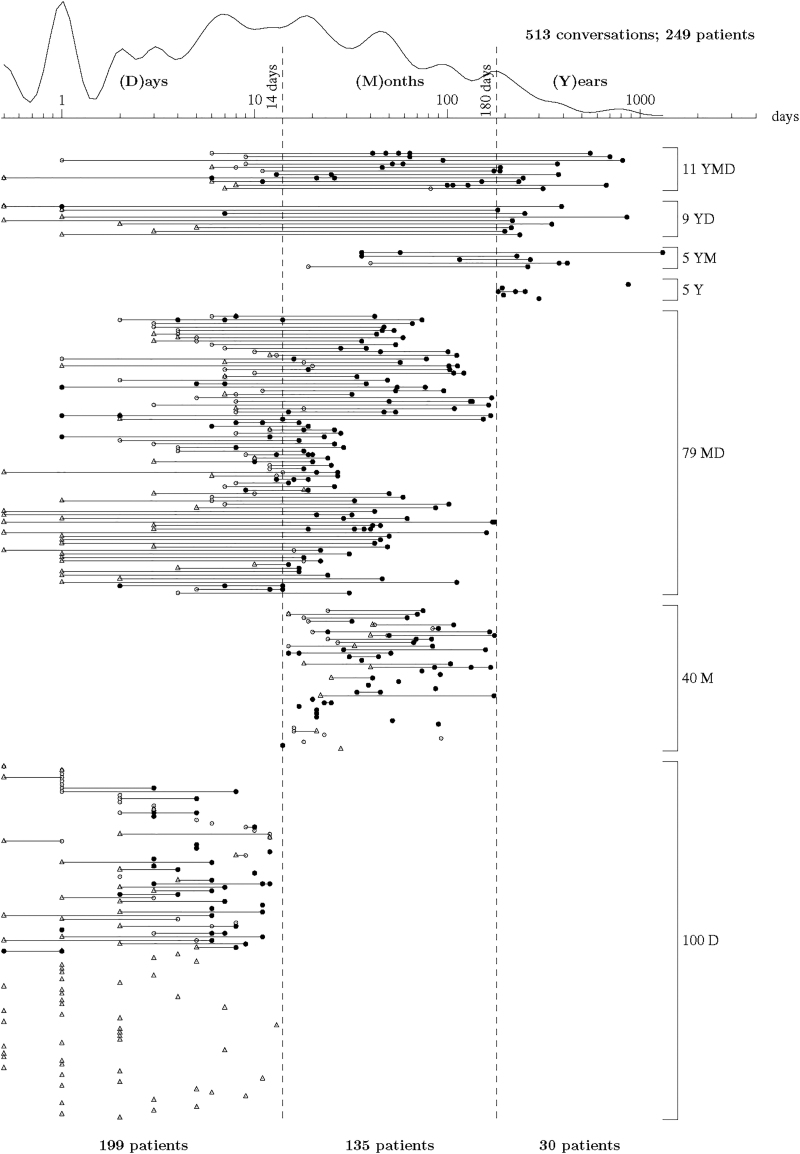
A view of all documented conversations in the medical records, over years, months, and days before death. On top, there is a frequency graph of the timing of conversations.

Using the years, months, and days framework, 12% of the patients had their first conversations more than six months before death. A larger proportion (46%) of the patients had their first conversations 6 months to 15 days before death, and 23% of the patients had their first conversation within 14 days of death. Four percent of the patients had conversations in all three time windows and 36% of the patients had conversations in two time windows (the majority at days/months, Fig. 2).

Two-thirds of the patients who had a conversation in the years time window also had a conversation within 14 days of death, and two-thirds of the patients who had their first conversation in the months time window also had conversations within 14 days of death.

### Conversations about dying

Using the medical records, we could verify a conversation about dying in 108 patients and an additional 48 patients with whom the conversation about dying was held with their next of kin, without the patient, out of 249 reported in the SRPC. This leaves 93/249 patients (37%) for whom we were unable to identify a conversation about dying in the medical records.

A review of conversation content among the entire study population (*N* = 249) revealed 513 documented conversations about serious illness. Of these, 245/513 (48%) were about dying (in some cases, the patient and their next of kin had separate conversations).

Among the patients who had a conversation about dying documented in the medical record, 76% had an earlier record entry regarding a conversation about serious illness, on average 49 days before death. Among the patients who did not have a conversation about dying recorded in the medical records, a slightly smaller proportion (65%) had at least one documented conversation about serious illness 39.5 days before death on average (data not shown).

### Time to death by care unit

Conversations about dying with patients occurred on average eight, four, and two days before death in specialized palliative care, oncology care, and COPD care, respectively (Table 2). More patients in specialized palliative care had a conversation about dying (54%) than those in oncology (24%) or internal medicine (23%).

**Table 2.** Data on Conversations About Dying at the Care Units, Based on Review of the 249 Medical Records

	*N* (%)	Days from conversation about dying to death: median/range
Specialized palliative care unit		
No. of patients	160	
Patient had a conversation about dying	87/160 (54%)	8/0–93
Next of kin had a separate conversation about dying	70/160 (44%)	3/0–41
Oncology ward		
No. of patients	49	
Patient had a conversation about dying	12/49 (24%)	4/1–23
Next of kin had a separate conversation about dying	30/49 (59%)	2/0–12
Internal Medicine Ward		
No. of patients	40	
Patient had a conversation about dying	9/40 (23%)	2/0–9
Next of kin had a separate conversation about dying	37/40 (90%)	1/0–8

The next of kin had conversations about dying without the patient present in a higher proportion in internal medicine (90%) and oncology (59%) than in specialized palliative care (44%).

## Discussion

In this study, we developed a better understanding of the continuum of communication about serious illness by examining medical records from the time of diagnosis of the main disease until death. We observed that the process of communication about serious illness takes place over years, months, and days. Most conversations occurred during the months and days before the very end of life, with 31% of the patients having both “months” and “days” conversations. However, very few patients (4%) had documented conversations in all three time windows.

Few conversations in our study occurred in the years time window, more than six months before death. This is consistent with a previous study from the United States that found the median first conversation about end-of-life care to be 33 days before death,^9^ with more than half of the conversations taking place in acute admissions as opposed to during outpatient care. This contrasts with current oncology guidelines that recommend conversations take place early, when life expectancy is years to months.^10,11^ Programs such as the Serious Illness Care Program, which focuses on clinician communication skills training and systems change, have been shown to increase the frequency of earlier conversations.^12^

However, it is unclear when these conversations should begin. When healthy people older than 65 years were asked to imagine that they had a serious illness and then consider when they would want to be informed of their prognosis, only 44% wished to be informed of a two-year prognosis. In contrast, 62% wished to be informed at one year, and 74% at six months. It became more important to engage in serious illness communication as death became nearer.^13^

Many conversations in our study took place within the “months” time window. In the months before death, death is not imminent. Thus, patients have the opportunity to consider options and make informed treatment decisions based on their values and priorities. Conversations in the “months” time window are associated with less intense end-of-life medical care.^14^

Our review of medical records showed that conversations about dying took place overall at a median of seven days before death, and eight days before death in the specialized palliative care unit. Eight years ago, a Swedish study showed that the median time between conversations about dying and death in a specialized palliative care unit was four days.^15^ The authors pointed out that even though the importance of having a conversation about dying had been promoted in national care programs in Sweden, conversations took place too late. Four days does not provide sufficient time for the patients and families to prepare for death. Our study shows improvement.

In our study, oncology ward patients still had a short time between their last conversation and death (four days). Several factors could explain this finding. For example, time constraints are often cited as a barrier to end-of-life communication, and inpatient oncology capacity has in fact been reduced by 30% during the last 20 years in southern Sweden, whereas the population has increased by almost 30% during the same years.^16–18^ Furthermore, cancer patients with poor prognoses in Sweden typically have earlier conversations about dying and then transfer to specialized palliative care, whereas cancer patients with uncertain prognoses are kept in the oncology ward.

Patients with COPD had conversations about dying only two days before death. This short time period may reflect that, while their frailty was acknowledged, their death still occurred quickly and relatively unexpectedly.^19^ This may also reflect a lack of communication skills training or a culture that does not promote early conversations. Discontinuity in patient-provider relationships is another possible reason why conversations in this patient population occurred close to death.

A conversation about dying, registered in the SRPC with the specification “documented in the medical records,” could only be verified in 63% of the patients' medical records, that is, 37% of the patients in the SRPC had incorrect information, at least when using the medical record as the gold standard. Medical records have been considered the gold standard for the validation of quality indicators in other studies^20^; however, in this instance, they may not accurately reflect clinical practice. Previous studies have found significant deficiencies in the documentation of end-of-life conversations, which can be regarded as errors of omission.^21,22^

In such cases, clinicians have conversations with patients and families, but fail to document them, or it may be that health care personnel misinterpret which transitions the SRPC intends to measure (Fig. 1). Similar confusion has been shown in comparable contexts: stakeholders cannot reach a consensus definition of Serious Illness Communication, which makes it difficult to apply measures.^23^

### Study limitations

Our study has several limitations. First, the relatively small sample size from a specific region limits our ability to generalize the findings. While this region can be argued to be representative of Sweden,^24^ the result may not apply to different countries with different processes for reporting end-of-life conversations. Second, the subjective nature of interpreting clinical notes makes it difficult to categorize the conversation about dying. Although the two reviewers carefully assessed the content and negotiated to a consensus, conversations may have been mislabeled or missed. Finally, it is possible that crucial information reported to SRPC did not appear in the medical records. However, the SRPC clearly specifies that an affirmative response in the register requires a conversation about dying to be documented in the medical record by a physician.

## Conclusions

In conclusion, while our patients were preselected for having had a conversation about dying, we found additional, earlier conversations about serious illness. In this study, we observed a pattern of conversations over years, months, and days, which we believe will be a helpful framework for clinicians and patients, to ensure sufficient information to prepare for the end of life. In addition, this framework may be a useful way to measure the quality of the process of communication about serious illness, and thus improve it.

## Data Availability

The authors will provide access to the de-identified data on request.

## Abbreviations Used

COPD: chronic obstructive pulmonary disease
SRPC: Swedish Register for Palliative Care
